# Characterization of age‐related penile microvascular hemodynamic impairment using laser speckle contrast imaging: possible role of increased fibrogenesis

**DOI:** 10.14814/phy2.13481

**Published:** 2017-11-09

**Authors:** Seung‐Ryeol Lee, Ki‐Ho Kim, Ho‐Song You, Johnny Fu, Tung‐Chin (Mike) Hsieh, Valmik Bhargava, M. Raj Rajasekaran

**Affiliations:** ^1^ Departments of Urology and Medicine VA San Diego Health Care System University of California San Diego California; ^2^ Department of Urology CHA Bundang Medical Center CHA University Seongnam Korea; ^3^ Department of Urology Dongguk University College of Medicine Gyeongju Korea; ^4^ Department of Urology Chonnam National University Hospital Gwangju Korea

**Keywords:** Erectile dysfunction, penile blood flow, penile blood microcirculation, penile blood perfusion

## Abstract

Current technology for penile hemodynamic evaluations in small animals is invasive and has limitations. We evaluated a novel laser speckle contrast imaging (LSCI) technique to determine age‐related changes in penile microvascular perfusion (PMP) and tested the role of cavernosal muscle (CC) fibrosis mediated by Wnt‐TGF *β*1 signaling pathways in a mouse model. Ten young (2–3 months) and old (24–28 months) wild‐type C57BL6 male mice were subjected to PMP measured using a LSCI system. Penile blood flow (PBF, peak systolic velocity, PSV) was also measured using a color Doppler ultrasound for comparison. Measurements were made before and after injection of vasoactive drugs: prostaglandin E_1_ (PGE_1_) and acetylcholine (ACh). CC was processed for immunohistochemical studies for markers of endothelium and fibrosis. Protein levels were quantified by Western blot.PMP and PBF increased significantly from baseline after injection of vasoactive drugs. Peak PMP after PGE_1_ and ACh was higher in young mice (225.0 ± 12.0 and 211.3 ± 12.1 AU) compared to old (155.9 ± 7.1 and 162.6 ± 5.1 AU, respectively). PSV after PGE_1_ was higher in young than old mice (112.7 ± 8.5 vs. 78.2 ± 4.6 mm/sec). PSV after ACh was also higher in young (112.7 ± 5.6 mm/sec) than older mice (69.2 ± 7.1 mm/sec). PMP positively correlated with PSV (*r* = 0.867, *P* = 0.001). Immunostaining and Western blot showed increased protein expression of all fibrosis markers with aging. LSCI is a viable technique for evaluating penile hemodynamics. Increased cavernosal fibrosis may cause impaired penile hemodynamics and increased incidence of erectile dysfunction in older men.

## Introduction

Erectile dysfunction (ED) is a common sexual problem among older men, and its prevalence increases with age. Pathophysiology of ED is multifactorial and includes vascular, neurogenic, endocrine, iatrogenic, and psychogenic causes (Lue 2000). ED can be considered as an extension of peripheral endothelial dysfunction because normal penile erection requires coordinated arterial endothelial‐dependent vasodilation and sinusoidal endothelial‐dependent corporal smooth muscle relaxation (Bivalacqua et al. 2003). Thus, vascular risk factors of endothelial dysfunction such as diabetes, hypertension, hypercholesterolemia, metabolic syndrome, and aging are closely associated with ED (Costa and Virag 2009).

Penile erection is a hemodynamic process and normal erectile function depends on maximizing arterial inflow while minimizing venous outflow (Meldrum et al. 2014). To evaluate penile hemodynamic function, Doppler ultrasonography (DUS) is clinically used to investigate penile macrovascular function, namely arterial inflow and veno‐occlusive dysfunction by measuring the penile blood flow (PBF) of cavernosal arteries and dorsal veins (Altinkilic et al. 2004). Most useful parameters of DUS are as follows: peak systolic velocity (PSV), end diastolic velocity (EDV), and resistance index (RI). Commonly used vasoactive agents during penile DUS, such as papaverine or prostaglandin E_1_ (PGE_1_), act directly on cavernosal smooth muscle relaxation by activating primarily cyclic adenosine monophosphate (cAMP)‐dependent pathways (Bivalacqua et al. 1999). Limitations of DUS include (1) variability in measurement of hemodynamic parameters due to operator dependence and (2) its limited potential to evaluate penile microcirculatory changes.

Arterial network is composed of macro‐ and microcirculation, which interact to enable an optimal adaptation to various physiologic disturbances. With age and/or other risk factors, modifications appear in both the macro‐ and microcirculation (Feihl et al. 2009). Microcirculatory disturbances have been postulated to be factors producing tissue damage in ED patients with vasculogenic cause. Monitoring large vessels' characteristics using DUS provide a good vital biomarker to assess the status of penile macrocirculation. Conversely, microvascular blood flow assessment is important for follow‐up of aging as well as vasculogenic pathologies such as diabetes but also to understand the impact of aging (Bentov and Reed 2015).

Recently, laser speckle contrast imaging (LSCI) has proven to be beneficial in evaluating systemic microvascular endothelial function in patients with cardiovascular diseases (Cordovil et al. 2012). It has been tried clinically in evaluating microvascular perfusion changes of burn wound and cerebral blood flow during neurosurgery (Richards et al. 2014; Mirdell et al. 2016). These observations suggest that it is possible to measure PMP and evaluate penile endothelial dysfunction using the LSCI.

We hypothesize that PMP measured with LSCI may be a viable noninvasive, operator‐independent technique to identify microcirculatory changes in small animal model. Thus the aims of our study were to (1) evaluate LSCI for assessing penile microcirculation; (2) compare age‐related penile macro (using DUS)‐ and microcirulatory (by PMP) changes in mice; and (3) determine if aging is associated with increase in penile fibrotic molecules by quantification of these markers (collagen‐I, axin‐1, *β*‐catenin, periostin, and TGF‐*β*1) in a mouse model.

## Materials and Methods

The institutional animal care and use committee at the VA San Diego Healthcare Systems approved the study protocol and all experiments were conducted in accordance with the Guide for the Care and Use of Laboratory Animals (National Institutes of Health, Bethesda, MD). Ten each of wild‐type C57BL6 male young (2–3 months) and old (22–24 months) mice were subjected to the physiological studies. At the end of the study, animals were sacrificed and cavernosal tissue harvested for immunostaining and Western blot studies. The following antibodies were used: Collagen‐1 (cat# ab90395, Abcam), Active *β*‐Catenin (clone 8E7, cat# 05‐665 Millipore), CD 31 (PECAM‐1; cat# 553370, BD Pharmigen), Periostin (cat# SC‐398631,Santa Cruz Biotechnology), TGF‐*β*1 (cat# SC‐130348, Santa Cruz Biotechnology), Axin‐1 (SC‐293190, Santa Cruz Biotechnology).

### LSCI for PMP measurement

**Figure 1:**
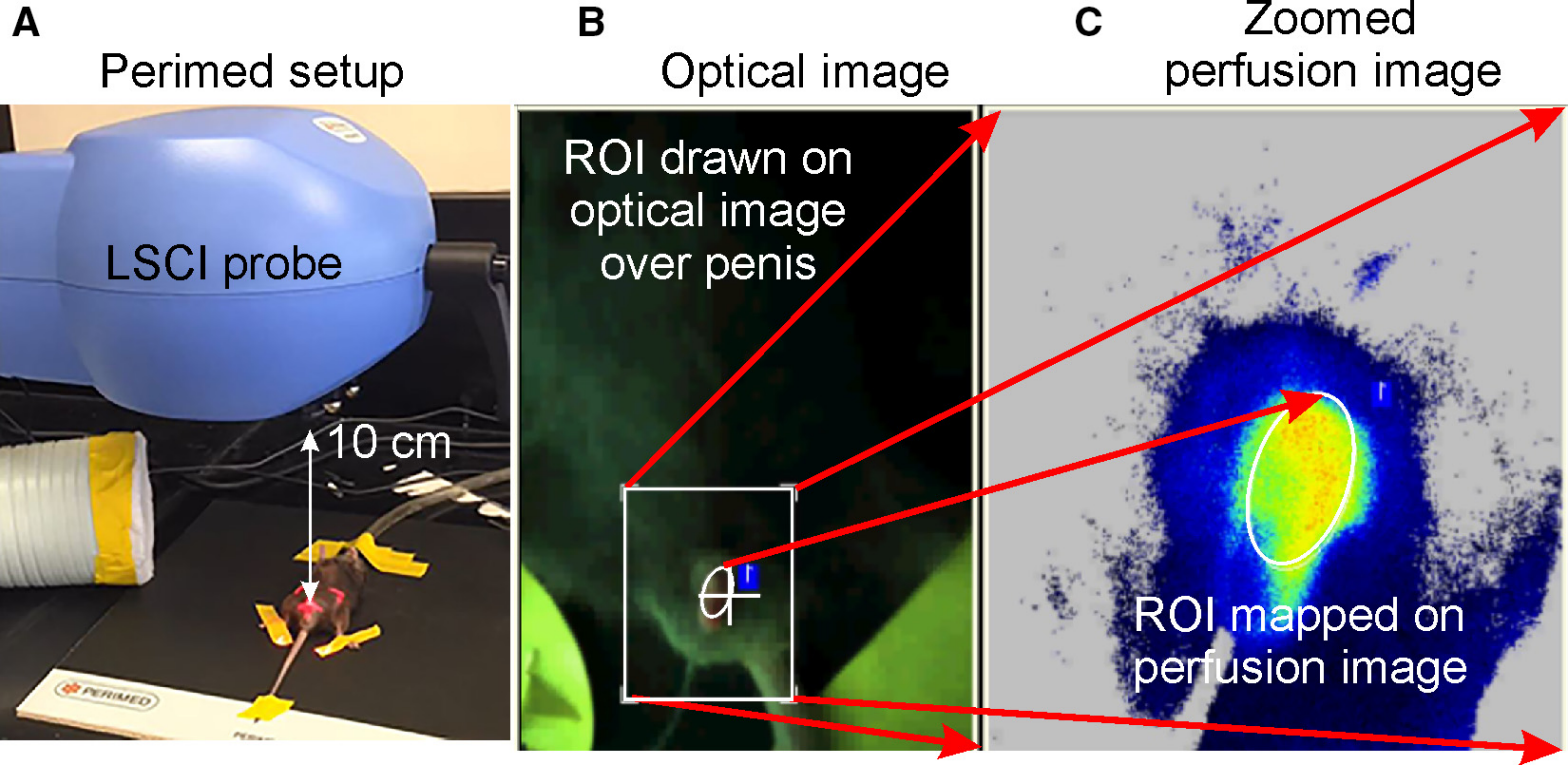
Experimental setup showing LSCI probe (A) focused on ROI, optical image (B), and perfusion image (C).

PMP was assessed by PeriCam PSI system (Perimed AB, Järfälla, Sweden). This technique quantifies PMP in arbitrary perfusion units (AU) and displays a real‐time perfusion image (Fig. 1). Mice were anesthetized with 5% isoflurane and placed in a supine position. Penis was carefully exposed and a 30‐gauge needle connected to a syringe with a polyethylene‐50 catheter tubing was inserted into the corpus cavernosum (CC). Scan head was positioned 10 cm above the penis. An elliptical region of interest (ROI) was selected for calculation of average perfusion (Fig. 1). Image sampling frequency was 19/s, and one average image/s was acquired. PMP measurements were made before and after intracavernosal injection (ICI) of: PGE_1_ (50 ng) as well as acetylcholine (ACh, 10 *μ*g). Studies using PGE_1_ and ACh were performed on different days. PMP was continuously acquired starting from the baseline until peak perfusion was reached after ICI of drugs, and it gradually decreased. PIMSoft 1.5 version was used for image analysis. Baseline and peak PMP and rise time (time to increase PMP from 20% to 80% of peak relative to baseline) were measured.

### DUS for PBF measurement

The Vevo3100 color Doppler system (FUJFILM VisualSonics Inc., Toronto, ON, Canada) with a 32–56 MHz linear probe was employed to measure PBF in the mouse. Mice were anesthetized and prepped as previously described. Heart rate was continuously monitored (range: 560–580 beats per minute) and maintained in the physiological range. A linear DUS probe was placed longitudinally at the lateral aspects of penile base. PBF was measured before and after ICI of PGE_1_ (50 ng) as well as ACh (10 *μ*g). Continuous wave Doppler images were digitally recorded. PSV was measured in young and old mice by placing a sample volume to be optimally oriented, such that the cavernosal artery is oriented as parallel to the ultrasound beam as possible. The ultrasound machine makes a correction for the difference in the angle between the ultrasound beam and vessel flow direction to calculate the blood velocity.

### Immunostaining studies

We performed immunofluorescence (IF) studies to localize endothelial (PECAM‐1) and fibrogenic markers: collagen‐1, axin, *β*‐catenin, periostin, and TGF‐*β*1. Cryocut tissue sections (10*μ*) were processed for antigen retrieval. Sections were incubated for 30 min with 5% normal goat/horse serum containing 1% Triton X‐100 to block the nonspecific binding sites. Sections were further incubated overnight at 4°C with specific monoclonal antibodies (1:200) dissolved in the PBS containing 1% serum. In one set, tissues were incubated with normal mouse IgG in the absence of primary antibody, which served as a negative control. After three washings, sections were further incubated for 2 h with appropriate anti‐mouse secondary antibodies and conjugated with rhodamine or FITC. Incubation was terminated by washing with PBS, and the slides were mounted in Gel/Mount. Slides were kept in the dark at 4°C and observed under a fluorescent microscope for imaging.

### Western blot studies

To quantify levels of fibrogenic markers (collagen‐1, axin, *β*‐catenin, periostin and TGF‐*β*1), the samples were homogenized with lysis buffer: [20 mmol/L Tris (pH 7.8), 137 mmol/L NaCl, 2.7 mmol/L KCl, 1 mmol/L MgCl2, 1% Triton X‐100, 10% glycerol, 1 mmol/L EDTA, 1 mmol/L dithiothreitol]. Samples were centrifuged at 600–800 g at 4°C and supernatant was collected. *β*‐mercaptoethanol and sample buffer were added to samples and heated at 100°C for 10 min. Samples were then loaded into 10% polyacrylamide precast gels and run at 140 V for 1.5 h. Membranes were blocked in tris‐buffered saline/Tween (1%) containing 3% BSA and incubated with primary antibodies (1:1000) overnight at 4°C. After washing, membranes were incubated for 2 h in a secondary antibody (1:3000). Blots were developed by enzyme chemiluminescence (ECL) method (Amersham). Blots were quantitated using GADPH to normalize densitometry values (Wilkes et al. 2004; He et al. 2010).

### Statistical analysis

Data are expressed as mean ± SEM and analyzed using Student's *t*‐test (using a one‐tail) or one‐way ANOVA, using *Graphpad Prism* (GraphPad, La Jolla, CA). *P* < 0.05 was considered significant. Where multiple comparisons were attempted, Bonferroni correction was made.

## Results

### Age‐related PMP changes

**Figure 2:**
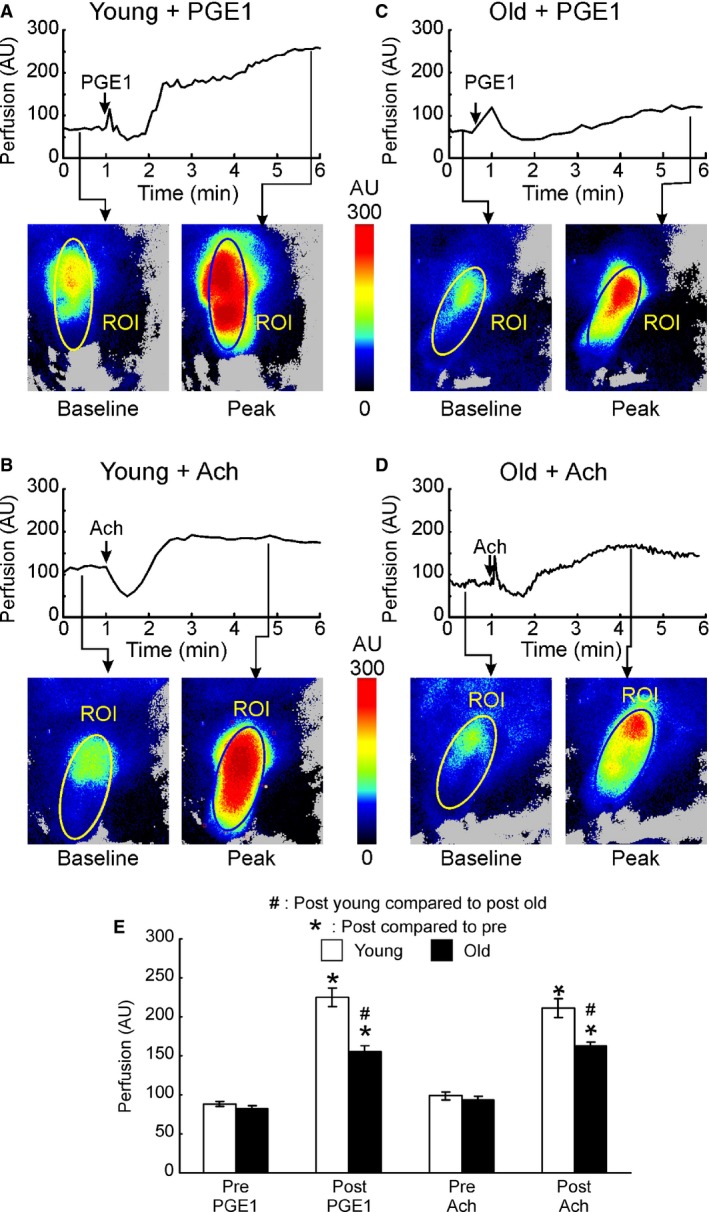
Representative line graph and LSCI of mice penis. Line graphs show the PMP from 1 minute before injection of vasoactive drug to 5 min after injection of vasoactive drug. Perfusion images show increased blood perfusion of ROI at peak (yellow and red) compared with that at baseline (blue and green). (A) Young mice with PGE_1_; (B) Young mice with Ach; (C) Old mice with PGE_1_; (D) Old mice with Ach; (E) Comparison of penile microvascular perfusion between young (white bars) and old (black bars) after an intracavernosal injection of vasoactive drugs (PEG_1_ and ACh).

Representative tracings from young and old mice showed stable PMP at baseline. PMP increased after ICI of PGE_1_ as well as ACh and reached peak (Fig. 2A). As PMP measurements PMP progressed from baseline to peak, the ROI colors changed from blue or green into yellow and then to red (Fig. 2A–D). ROI colors in young mice (Fig. 2A and B) showed compared significantly greater increase compared with old mice (Fig. 2C and D). There was no significant difference in baseline PMP between young and old mice (Fig. 2E). Injection of PGE_1_ as well as ACh produced a significant increase in PMPs in both young and old mice (*P* = 0.001, Fig. 2E). However, the peak PMP after PGE_1_ was significantly higher in young compared to old (*P* = 0.001, Fig. 2E). A similar trend in peak PMP was observed after ACh as well (*P* = 0.003, Fig. 2E). There were no significant differences in time taken to achieve the peak PMP between young and old mice (data not shown).

### Age‐related PSV changes

**Figure 3:**
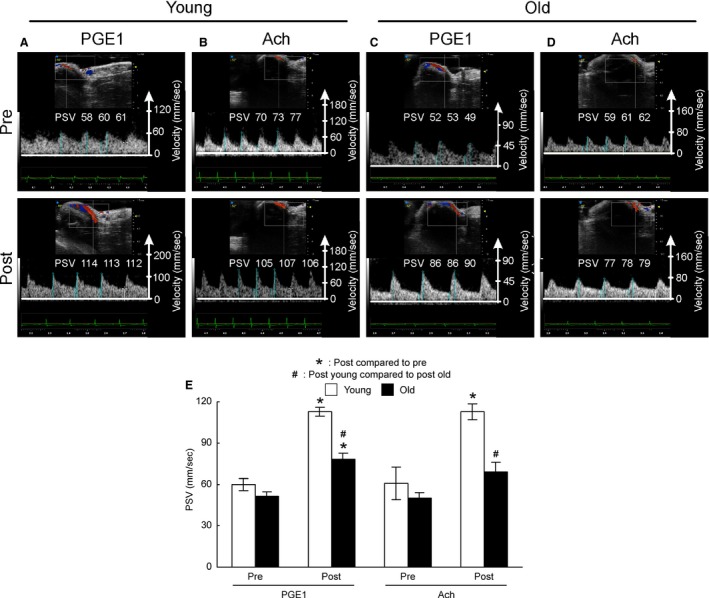
Representative Doppler blood flow images. (A) Young mice with PGE_1_; (B) Young mice with Ach; (C) Old mice with PGE_1_; (D) Old mice with Ach; (E) Comparison of PSV of young (white bars) and old (black bars) after an intracavernosal injection of vasoactive drugs (PEG_1_ and ACh). PSV was obtained by averaging three measurements for each animal.

Representative PSV tracings in young and old mice before and after ICI of PGE_1_ as well as ACh are shown in Figure 3A–D. There was no significant difference in baseline velocity between young and old (Fig. 3E). ICI of PGE_1_ as well as ACh elicited a significant increase in PSV in both young and old mice (Fig. 3E). However, PSV of young mice was significantly higher than older animals after ICI of PGE_1_ (*P* = 0.001). A similar trend in peak PSV was observed after ACh as well (*P* = 0.003).

### Correlation between PMP using LSCI and PSV using DUS

**Figure 4:**
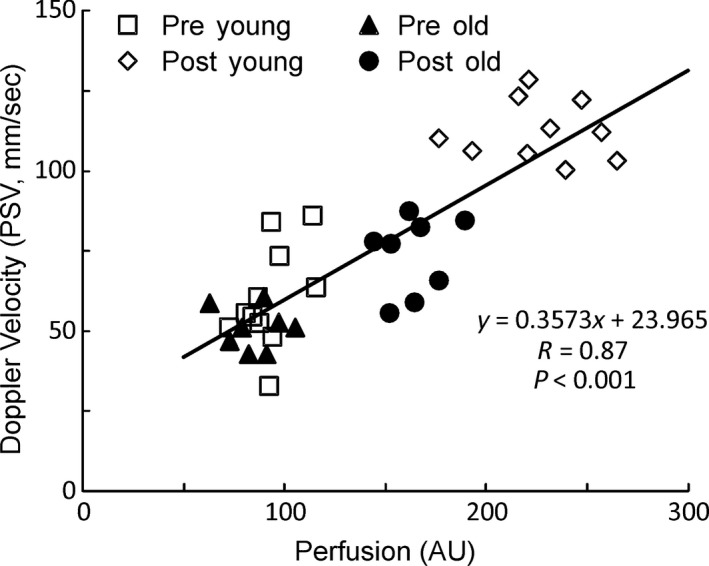
Correlation between penile microvascular perfusion (PMP) and penile blood flow (PBF‐PSV). PMP correlated positively with PBF (correlation coefficient = 0.867, *P* = 0.001).

Baseline and peak PMP after ICI of both vasoactive agents were paired with baseline flow and PSV, respectively. A significant positive correlation of PMP with PSV was observed (*r* = 0.867, *P* = 0.001, Fig. 4).

### Immunofluorescence studies

**Figure 5:**
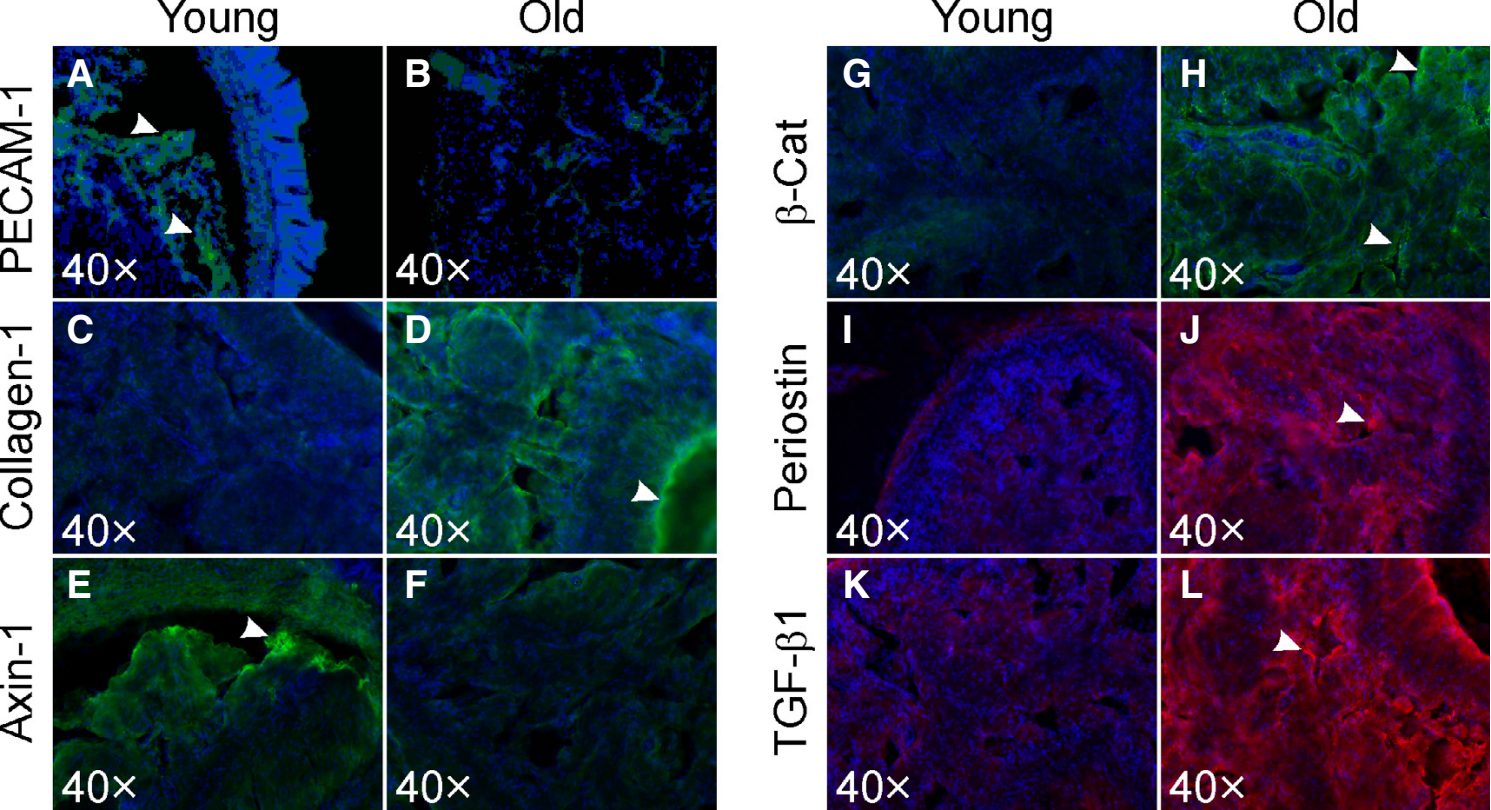
Representative fluorescence images showing immunolabeling for PECAM‐1, collagen‐1, axin‐1, *β*‐catenin, periostin, and TGF‐*β*1 in penile tissue cross sections (within cavernosal sinusoids) from young and old mice. PECAM‐1, collagen‐1, axin‐1, *β*‐catenin (green), periostin, and TGF‐*β*1 (red) are shown. Localization of all markers mostly within cavernosal sinusoids are seen. Intense staining for all markers except PECAM‐1 and axin‐1 in old mice (white arrow heads; D, H, J, L). Intense staining for PEACM‐1 and axin‐1 is seen in penile cross sections from young mice (A, E).

Figure 5 shows localization of PECAM‐1, collagen‐1, axin‐1, *β*‐catenin, periostin, and TGF‐*β*1 in young and old mice cavernosal tissue. Immunolabeling of PECAM‐1 (A‐B) was observed around the endothelial lining whereas the fibrogenic protein labeling (collagen‐1, axin‐1, *β*‐catenin, periostin, and TGF‐*β*1; panels C‐D; E‐F; G‐H; I‐J; K‐L) was observed throughout the cavernosal smooth muscle. An intense staining for PECAM‐1 was noticed in sections from young mice (A). In aging penis, a noticeable increase in all fibrosis markers (C, G, I, K) except axin‐1 was observed. Conversely, an intense localization for axin‐1 (E) was seen in young penis (F).

### Western blot analysis

**Figure 6:**
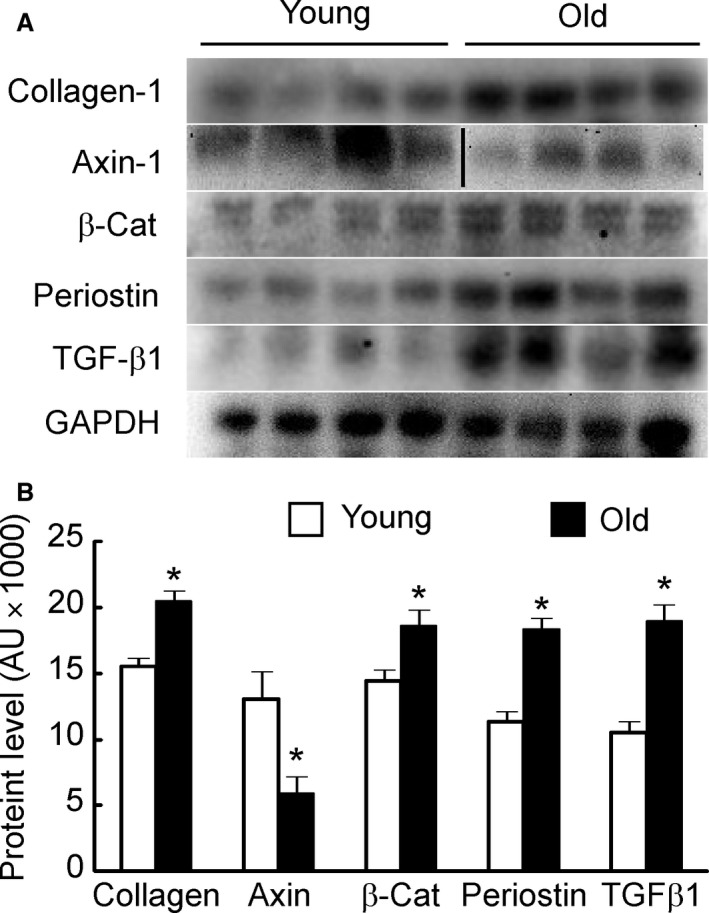
Panel A shows Western blot gels for fibrotic markers in cavernosal tissues from young and old mice. Panel B shows a significant increase in all markers, except axin, in old mice penis compared to the young (after normalization with GAPDH). * *P* < 0.05.

Figure 6A shows representative images depicting age‐related changes in protein levels of the five fibrogenic proteins, collagen‐1, axin‐1, *β*‐catenin, periostin, and TGF‐*β*1. Image analysis revealed a significant change in all fibrogenic proteins (Fig. 6B). Major changes (1.5‐ to 2‐fold increase) were observed in axin‐1, periostin, and TGF‐*β*1 levels in old mice samples than those from young.

## Discussion

Our study aims were to (1) evaluate the potential of LSCI for assessing PMP; (2) compare the PMP with DUS to evaluate age‐related penile macro and microcirulatory changes in old male mice; and (3) determine if aging is associated with increase in the molecules known to be associated with fibrosis. We used LSCI and DUS to measure PMP and PSV, respectively. Our studies showed age‐related impairment of PMP and a very good correlation with clinically used PSV.

Currently, intracavernosal pressure (ICP) and penile DUS have been used as the “gold” standard diagnostic parameters to evaluate ED in preclinical and clinical studies, respectively. However, DUS has its limitations. In addition, the knowledge gained from ICI of PGE_1_ alone may be inadequate to demonstrate endothelial dysfunction (Moreland et al. 2001; Nehra et al. 2002). Endothelial dysfunction is now recognized to be a common cause of ED, which partially explains the links between ED and vascular disease beyond the penis. DUS evaluation with PGE_1_ had a very limited accuracy in discriminating ED in the presence of vascular risk factors (Bocchio et al. 2006). To evaluate penile endothelial function and to overcome these limitations, the shear‐stress flow‐mediated vasodilation of the cavernous arteries was measured by DUS (Virag et al. 2004; Mazo et al. 2006). The internal diameter of one of the cavernous arteries was measured before and after an occlusion of the PBF by inflating an occlusion cuff placed at the penis base because a reactive vasodilatation mediated by the vascular endothelium takes place when the artery is occluded and then unoccluded (Joannides et al. 1995). However, since it has been shown that site as well as duration of cuff occlusion could influence the outcome, similar technical issues may hamper accurate measurements that may affect exact diagnosis (Agewall et al. 2001; Betik et al. 2004; Sejda et al. 2005).

The microcirculation refers to arteries with the smallest resistance (less than 50 nm in diameter), arterioles, capillaries, and venules. In our study, we used both PGE_1_ and ACh for PMP as well as PSV measurements. PGE_1_ acts mainly directly on cavernosal smooth muscle through cAMP pathways, whereas ACh is an endothelial‐dependent vasodilator (Bivalacqua et al. 2003). PMPs of both young and old mice significantly increased after ICI of both PGE_1_ and ACh, but peak PMPs after ICI were significantly higher in younger than older mice. These results suggest that PMP can differentiate endothelium dependent as well as independent microvascular hemodynamic impairment in penis between young and old mice. In addition, we evaluated the correlation between the PMP and PBF and found that PMP positively correlated with the PBF. Finally, our results suggest that PMP can be a useful noninvasive, operator‐independent diagnostic tool for the assessment of ED.

To evaluate the erectile function in animals, measuring intracavernosal pressure (ICP) has been the standard technique (Piao et al. 2007; Zhao et al. 2009). However, this technique is invasive, terminal and therefore longitudinal monitoring of penile hemodynamics is not possible. In addition, it needs measuring mean arterial pressure for normalizing because ICP is influenced by both penile vascular and systemic hemodynamics. On the other hand, measuring LSCI perfusion is easy, noninvasive, and can be measured several times for long‐term follow‐up, even in small animal models such as mouse. LSCI has been used to determine microcirculation in several tissues such as in bowel lesions in neonates (Knudsen et al. 2017), in brain to determine cerebral blood flow (Freitas et al. 2017), in renal cortical perfusion(Mitrou et al. 2016), retinal and skin perfusion (Senarathna et al. 2013).

Our immunostaining findings confirm endothelial dysfunction and fibrosis in the aging mouse penis. Protein levels of all the fibrosis markers except axin‐1 were increased in the aging penis. Conversely, axin‐1 levels were decreased in the old mice. Axin‐1 is a scaffolding protein involved in the regulation of Wnt‐*β*‐catenin signaling (Ulsamer et al. 2012). Lower levels of axin‐1 in aging penis suggest that there is dysregulation of Wnt‐*β*‐catenin pathways which may contribute to the increased fibrosis. There are several important signaling pathways that regulate muscle fibrosis. Among these, canonical Wnt/*β*‐catenin signaling and TGF‐*β* pathways are thought to be major players (Akhmetshina et al. 2012). Wnt/*β*‐catenin signaling is activated due to injury as well as during aging and is reported to play a key role in several types of fibrosis, that is, ischemia‐induced myocardial fibrosis, idiopathic, and bleomycin induced pulmonary fibrosis, fibrosis seen in chronic kidney failure, liver fibrosis, and abnormal skin wound healing (Fathke et al. 2006; Carre et al. 2010). In these examples, Wnt and positive regulators of *β*‐catenin signaling are upregulated and inhibitors of Wnt/*β*‐catenin signaling are downregulated. Akhmetshina et al. (2012) showed that canonical Wnt signaling is critical for TGF‐*β*‐mediated fibrosis and implicates a key role for the interaction of both pathways in the pathogenesis of fibrotic diseases. Our study results suggest that age‐related cavernosal muscle fibrosis involves both Wnt and TGF‐*β*‐mediated signaling pathways support these interactions between major fibrosis pathways. Axin‐1 is a scaffolding protein, and increased levels of this protein in young penis support its regulatory role in fibrosis (Ulsamer et al. 2012). Previous studies implicate a role for periostin in age‐related fibrosis (Chiao et al. 2012) and our findings confirm a role for periostin as a novel marker in penile fibrosis. Targeted antifibrotic therapy has been shown to reduce fibrosis and improve organ function (Bastakoty and Young 2016).

This study has some limitations. Our tissue analysis shows that profibrotic markers are increased but that does not prove the cause and effect relationship. Direct demonstration of fibrosis suppression after injection of specific pharmacological antagonists would prove the cause and effect relationship. We hope to confirm this in our future studies.

## Conclusions

Our studies show that PMP measured with LSCI is a viable noninvasive, operator‐independent technique to identify microcirculatory changes in small animal models. PMP positively correlated with PSV. These results further suggest that the PMP measured by LSCI is feasible for diagnosis of vasculogenic ED in men. Future clinical studies will be needed to validate our findings.

## Conflict of Interest

None declared.
